# Importance and impact of surveillance of the microbial quality of dialysate and RO water in a tertiary health

**DOI:** 10.1186/1753-6561-5-S6-P211

**Published:** 2011-06-29

**Authors:** M Sri Ratnamani, R Rao

**Affiliations:** 1 Microbiology, Apollo Hospitals Hyderbad India, Hyderabad, India

## Introduction / objectives

To find the source of bacteremia in patients undergoing haemodialysis in our hospital and to know the impact of surveillance on preventing dialysis related infections.

## Methods

This study was undertaken to find out the cause of bacteremias in patients undergoing haemodialysis in Medical Intensive Care unit (MICU) in our hospital.These patients developed fever ,leukocytosis post haemodialysis and Blood cultures were positive . A total of 140 samples consisting of 64 Reverse Osmosis( RO )water samples from RO Water port in MICU and Dialysis unit., 56 dialysate samples and 10 each of bicarbonate and acid concentrate solution from the hemodialysis unit were analysed.

## Results

Commonest isolate in dialysate was *Pseudomonas species* (45%) followed by *Citrobacter diversus* (28%) and *Acinetobacter species* (15%),while in RO water from MICU *Acinetobacter species* (39%) was the most common followed by *Pseudomonas species* .15 of the 64 RO water and 49 of the 56 dialysate had growth,. 3 of the 10 samples of acid concentrate showed growth .The bicarbonate concentrate tested showed high rate of bacterial contamination.. 10 RO water samples from MICU showed bacterial contamination ,Dialysate samples had higher colony count compared to the RO water samples. 16% of the Dialysate samples were non compliant with the AAMI RD 52 standard. 30 % of Bicarbonate concentrate were non compliant .The RO water samples tested from dialysis unit were 100% compliant with AAMI standards.

## Conclusion

The RO water contamination was due to absence of loop line in the MICU which led to stagnation in the RO water port . Regular microbiological monitoring of RO water and Dialysate is needed to ensure safety in patients undergoing haemodialysis.

## Disclosure of interest

None declared.

